# Spanish Cross-Cultural Adaptation, Rasch Analysis and Validation of the Ocular Comfort Index (OCI) Questionnaire

**DOI:** 10.3390/ijerph192215142

**Published:** 2022-11-17

**Authors:** Ana Rosa Barrio, Mariano González-Pérez, Clara Heredia-Pastor, Jacobo Enríquez-Fuentes, Beatriz Antona

**Affiliations:** 1 Optics and Optometry Department, Faculty of Optics and Optometry, Complutense University of Madrid, 28037 Madrid, Spain; 2 Applied Vision Research Group, Faculty of Optics and Optometry, Complutense University of Madrid, 28037 Madrid, Spain; 3 Hospital Clínico San Carlos, 28040 Madrid, Spain

**Keywords:** Ocular Comfort Index (OCI), dry eye disease, symptoms, questionnaire, Rasch analysis, validation, Spanish population, translation, cross-cultural adaptation

## Abstract

The Ocular Comfort Index (OCI) assesses ocular surface irritation and grades the severity of dry eye disease. This study sought to adapt the OCI questionnaire into Spanish, and then to assess the psychometric performance and validity of the new adapted version (OCI-versión española, OCI_VE_). The questionnaire was translated, back translated, and then cross-culturally adapted for use with Spanish-speaking individuals. The OCI_VE_ was completed by 450 participants, including 53 subjects that were diagnosed with dry eye disease. Through a Rasch analysis, the psychometric properties of item fit, targeting, person separation, reliability, and differential item functioning (DIF) were assessed. To test the convergent validity, we examined the correlation between the OCI_VE_ and the Computer Vision Symptom Scale (CVSS17). Validity was tested in a subgroup of participants with and without dry eye, and test-retest repeatability was determined in a subset of 151 individuals. We also compared, via DIF, the performance of the OCI_VE_ with that of the original OCI. Our Rasch analysis revealed a good model fit, high accuracy, good targeting, unidimensionality, and no DIF according to gender. The validity and repeatability were good. The OCI_VE_ shows comparable psychometric properties to the original English version, making it a valid tool for measuring dry eye symptoms in Spanish adults.

## 1. Introduction

Dry eye disease (DED) is a multifactorial disorder that especially affects older persons and menopausal women [1], and it is a frequent motive for visiting a family care provider or ophthalmologist. In a review by TFOS Dry Eye Workshop (DEWS) II of prevalence studies conducted over the period from 2005 to 2015, reported DED prevalence ranged from 8.7% to 30.1% in studies where symptoms and signs were combined [2]. In Spain, its prevalence in the general adult population aged ≥ 40 years was estimated at 11%, and it was found to be more frequent in women (11.9%) than men (9%), as well as significantly associated with age [3]. Apart from female sex and advanced age, other factors known to increase the risk of DED are Sjogren’s syndrome [4], meibomian gland dysfunction [5,6], prolonged VDT use [7,8], contact lens wear [9,10,11], and some medications, such as antihistamines, antidepressants, or anxiolytics. In addition, several environmental factors have been also shown to provoke DED, such as air pollution [12,13], wind, low humidity [14], and high altitude [15,16]. In effect, the TFOS DEWS II Epidemiology Report suggested a need for studies designed to identify further environmental factors that could trigger DED [2]. DED has significant repercussions on quality of life, so it is important to recognize the symptoms and signs of dry eye so that treatment can be promptly started. While the diagnosis of DED is usually based on a medical history and physical examination, symptoms and signs of DED can be inconsistent in some patients [17,18,19].

In 2007, DEWS classified dry eye as: “a multifactorial disease of the tears and ocular surface that results in symptoms of discomfort, visual disturbance, and tear film instability with potential damage of the ocular surface. It is accompanied by increased osmolarity of the tear film and inflammation of the ocular surface”. In this new definition of dry eye, symptoms become more important as a characteristic of the disease, contrasting with the previous definition, which focused more on visible signs of ocular surface damage and tear film dysfunction [20].

Accordingly, questionnaires of ocular surface-related signs and symptoms have recently taken on a relevant role in the diagnosis and management of patients with dry eye [21,22]. These questionnaires enable clinicians to diagnose and determine the severity of dry eye to manage and monitor this condition. They can also be used as screening tests, as they can detect cases of dry eye that are also subclinical or unrecognizable [23]. The PRO guidance document, released by the U.S. Food and Drug Administration (FDA), states that a clinical questionnaire should have satisfactory levels of psychometric properties before its use in clinical practice [24,25].

The Ocular Comfort Index questionnaire (OCI), developed for English speakers by Johnson and Murphy in 2007 [26] through Rasch analysis, serves to quickly assess dry eye symptoms. This questionnaire has proven and robust psychometric properties [24,27] and provides valid reliable measurements [26,28]. The OCI shows moderate positive correlation with the Ocular Surface Disease Index (OSDI) [26], yet its scores are only weakly and negatively correlated with tear breakup time (TBUT) [26,28], Schirmer I testing, and corneal fluorescein staining [28]. Khadka’s review provides a descriptive catalogue of ophthalmic patient-reported outcome (PRO) instruments to help researchers and clinicians select the most appropriate instrument for its purpose. For dry eye and ocular surface disease, the most recommended higher-quality PRO instrument was OCI. This tool showed a superior performance to OSDI and McMonnies in terms of its content development, Rasch-based psychometric properties, validity, and reliability [27]. The OCI is suitable for use in clinical trials and allows for the assessment of the impact of ocular surface disease on patient well-being and of the effectiveness of specific dry eye treatments [26]. To date, there has been only one adaptation of the OCI. This recently developed Chinese version [29] of the questionnaire has psychometric properties comparable to those of the original version.

In the world, there are currently 493 million native Spanish speakers, and as the fourth most spoken language, the number of users of Spanish exceeds 591 million (7.5% of the world population) [30]. However, apart from the OSDI test, there are few validated questionnaires of ocular surface symptoms in Spanish. The objective of the present study was therefore to develop a valid Spanish version of the OCI questionnaire, and to test its psychometric performance through a Rasch analysis, along with its validity and repeatability.

## 2. Materials and Methods

Before the study outset, the authors of the OCI gave us their consent to develop a Spanish version of their instrument.

The OCI is composed of 12 items that assess the frequency and intensity of six symptoms (dryness, grittiness, stinginess, tiredness, pain, and itching) over the past week. Each item is scored on a scale from 0 to 6 (never to always, or absent to severe). According to the Rasch analysis, the total score becomes a linear continuous interval scale, which ranges from 0 (least symptomatic) to 100 (most symptomatic). A logit score and 0–100 scale is obtained by entering the results into the OCI calculator [26].

Rasch methods are based on the natural logarithm of odds ratios, and so measure both the item difficulty and person ability in log-odd units (logits). Each logit step increases the odds of observing the event specified by a factor of 2.72. This probabilistic method yields a logit score. A negative logit score indicates that the item is a below average difficulty, while a positive score indicates an above average difficulty. For a person’s ability, negative scores indicate below average ability, and the reverse is true for positive values [31].

The study was conducted in two stages: (1) the questionnaire was translated and cross-culturally adapted to Spanish; and (2) the Rasch-based psychometric properties, validity, and repeatability of the adapted version were assessed.

PARTICIPANTS

The study sample was recruited among the students and staff of the Optics and Optometry Faculty of the Universidad Complutense de Madrid, employees of the Havas Company, and patients of the Ocular Surface and Inflammation Unit of the Hospital Clínico San Carlos, Madrid, Spain. The study protocol adhered to the principles of the Helsinki Declaration and was approved by the Ethics Committee of the Hospital Clínico San Carlos (Madrid, Spain). Each of the 450 participants included gave their written informed consent after being informed of the nature and intention of the study.

The inclusion criteria were that participants were over 18 years of age. Exclusion criteria were: Spanish not being their native language, having had prior visual surgery (not refractive), having an active neurologic or visual disease apart from DED, using any medication that could affect vision, or having any kind of disability that could prevent the subject reading or understanding the questionnaire’s questions.

The questionnaire’s convergent validity was assessed in 257 participants who used digital display screens closely for at least 4 h a day. In addition, a subgroup of 151 of these participants completed the questionnaire at the study outset, and then again after a week to examine the repeatability of the questionnaire.

For the validity assessment, a subgroup of 53 healthy controls and 53 patients diagnosed with DED of varying severity were recruited from the hospital’s Ocular Surface and Inflammation Unit. The two groups were comparable in age and gender.

For the Rasch analysis of the original English version of the OCI and the subsequent comparison with the Spanish version, 216 participants, who fulfilled the same inclusion and exclusion criteria and lived in the United States of America (USA), also completed the questionnaire (mean age 40.21 ± 11.79 years; 53.24% female). All of them used digital display screens for 4 h or more a day. For this part of the study, we conducted a cross-sectional online survey on the research platform, developed and managed by Prolific Academic Ltd. Prolific (London, UK). This network connects researchers with individuals around the world who are interested in participating in online research studies [32].

**Figure 1:**
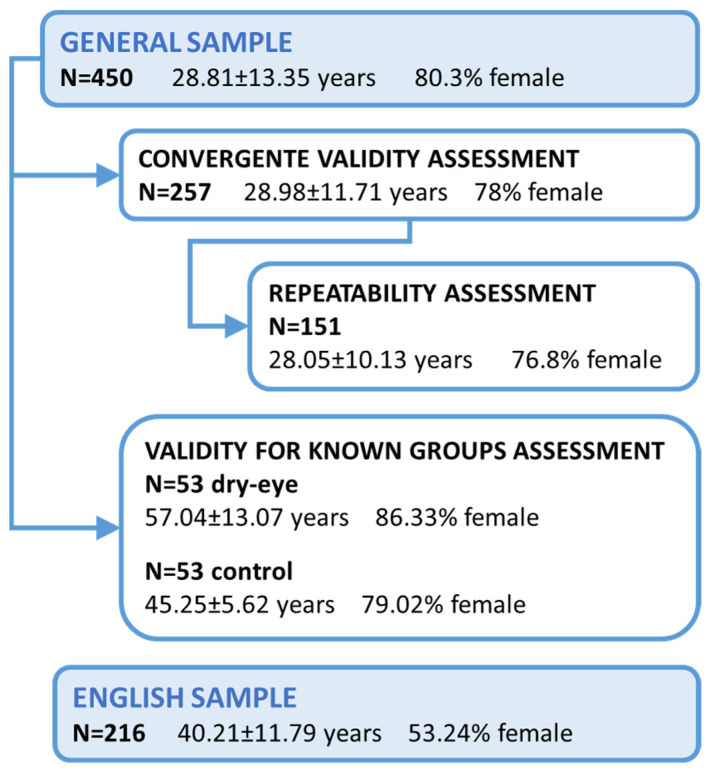
Description of the different samples used in the study.

Figure 1 describes the different samples used in our study.

### 2.1. Translation and Cross-Cultural Adaptation of OCI

The OCI was translated and adapted following the previously published guidelines [25,33,34,35] in a five-step process:

(1) Direct translation. Two bilingual (English/Spanish) translators, whose native language was Spanish, independently translated the original OCI questionnaire, including instructions, items, and grading options.
(2) Consensus version of the direct translation. The two bilingual translators agreed on the translated version and a template of the instrument was constructed.
(3) Back translation. Another two bilingual (English/Spanish) professional translators blind to the original version, whose mother tongue was English, independently translated the consensus version back into English.
(4) Expert committee review. The panel of experts, including the four translators, two expert optometrists, and a methodologist, compared the back-translated version with the original English version to identify any discrepancies to be resolved by consensus. They consolidated the four previous translations and created the pre-final version of the Spanish OCI designated OCI-versión española (OCI_VE_).
(5) Pre-testing of the consensus version. Interviews were conducted using a verbal probing technique with 30 native Spanish individuals aged between 18 and 70 years to ensure patient comprehension of OCI_VE_. The subjects completed the consensus version of the questionnaire, and then commented on words and sentences they considered difficult to understand. No issues emerged in this pre-test.

### 2.2. Rasch Analysis

The package Winsteps (Version 4.0.1, Winsteps, Beaverton, OR, USA) [36] was used for the Rasch analysis. To generate the descriptive data, we used the IBM SPSS Statistics package version 25.0 (Statistical Package for Social Sciences, IBM, New York, NY, USA).

We selected the Andrich Rating Scale Model (RSM), which assumes equal category thresholds across items, as all items share the same response option structure [37]. The quality of the psychometric data obtained at this stage was assessed according to the criteria proposed by Khadka et al. [27] for the quality assessment of ophthalmologic questionnaires. After eliminating participants who left more than 33% of questions unanswered, the variables assessed were:

- Item fit statistics. Infit and outfit were used to determine whether items fit the Rasch model.
- Dimensionality. Unidimensionality is a fundamental requirement of a measurement, implying that the scale measures a single concept [27]. This was determined by Principal Component Analysis (PCA) of the standardized residuals of the model using the Winsteps application. When the raw variance explained by the measures is ≤50%, multidimensionality is suggested. This indicates that the questionnaire may contain a subset of items that could be measuring a different concept.
- Person Separation Index (PSI) and OCI_VE_ levels of performance. PSI is a measure of the discriminating capacity of a questionnaire. A PSI of two is the minimum accepted level of discrimination for an instrument to produce a valid measure [38]. The number of different levels of performance was calculated, according to a method suitable for clinical samples described by Wright [29,39].
- Targeting. This refers to the extent to which the difficulty of the items matches the abilities of the persons; ideally, they should center on the same mean. This can be assessed visually by observing the person-item map, which is a graphical representation of persons and items on the logit scale. Differences > 1 logit indicate possible mistargeting.
- Differential Item Functioning (DIF). DIF assesses whether items are responded to differently by different population subgroups. We evaluated the DIF of each item by gender and presbyopia (male-female; presbyopia-no presbyopia) using the Mantel–Haenszel method and logit-difference (logistic regression) method implemented in Winsteps. A difference in difficulty of the item measure between the two groups was that: <0.50 logits was considered as no-DIF, minimal DIF as 0.50 to 1.0 logits, and notable DIF as >1.0 logits [36].

The sample size required to assess a questionnaire using a Rasch model was calculated according to Linacre criteria [40], whereby for a level of confidence of 99%, 20 *n of items would be needed. In our case, this was 240 subjects.

### 2.3. Validity and Repeatability

Convergence validity: The OCI_VE_ and Computer Vision Symptom Scale (CVSS17) [41] were administered online in random order to a subgroup of participants (N = 227). Convergent validity was assessed by estimating the coefficient of correlation between subjects’ OCI_VE_ and CVSS17 scores. According to Khadka et al. [27], a coefficient of correlation between the OCI and CVSS17 greater than 0.3 may be considered proof of convergent validity. Data were assessed for normality using the Shapiro–Wilk test. Due to the non-normal distribution of measures, Spearman’s rank correlation coefficient was calculated for comparisons. The significance was set at *p* < 0.05.

Validity for known groups: The OCI_VE_ was administered to a subgroup of 53 healthy controls and 53 patients diagnosed with different DED severity. The severity of dry eye in patients with DED and the absence of signs of DED in control patients were determined in a basic slit-lamp examination. Corneal and conjunctival staining were graded using the Oxford scale [42].

Repeatability: A subgroup of 151 participants completed the OCI_VE_ in two sessions, seven days apart. We then calculated the intraclass correlation coefficients (ICC) and within-subject standard deviations (Sw). The Bland–Altman method [43] was used to determine the mean difference (MD) between sessions, the coefficient of repeatability (CoR), and 95% agreement limits.

## 3. Results

### 3.1. Spanish Version of OCI (OCI_VE_)

Table 1 shows the items and response options of the original OCI and of the Spanish version (OCI_VE_), based on the pre-test administered to 30 individuals.

### 3.2. Rasch Analysis

OCI_VE_ was completed by 450 participants (mean age 28.81 ± 13.35 years; range, 19–87 years; females, 80.3%). After applying the inclusion and exclusion criteria and according to established criteria [44], 39 questionnaires showing an Outfit > 2.5, and one participant who left more than 33% of items unanswered, were excluded, so that 410 completed questionnaires were finally analyzed. The responses were subjected to Andrich’s Rating Scale Model (RSM) analysis implemented in Winsteps.

The mean OCI_VE_ score obtained was 34.41 ± 12.50 on a scale from 0 to 100 (M = −1.15; SD = 1.25 logit scale) and the range was from 2 to 70. No floor or ceiling response patterns were observed.

#### 3.2.1. Item Fit Statistics

The item fit statistics and item measures (difficulty in logits) obtained for the OCI_VE_ are provided in Table 2. The range of average item difficulties of the 12-item OCI was from −1.29 to 1.00 logits. More negative logit scores indicated less frequent and intense symptoms.

The fit statistics of most of the OCI_VE_ items (11 of 12 items) performed well. Only the infit of item 3 (1.40) was outside the more stringent criterion (0.7–1.3) proposed by Pesudovs et al. [45] and Khadka et al. [27].

#### 3.2.2. Dimensionality

Our PCA analysis of OCI_VE_ revealed that 67.3% of the raw variance was explained by the OCI_VE_ measures. As this variance was greater than 50%, we can consider the OCI_VE_ unidimensional.

#### 3.2.3. Person Separation Index and Performance Levels

The PSI for OCI_VE_ was 3.42, showing a reliability of 0.90. Cronbach’s α was 0.94. This means that the OCI_VE_ was able to distinguish 4.9 strata of scores, according to the equation [(4*PSI + 1)/3)]. However, according to the Wright method, which has the advantage of being independent of the sample and suitable for clinical samples, OCI_VE_ could distinguish 7.3 levels of symptoms.

Table 3 shows the correspondence between the OCI_VE_ raw score and level of performance.

#### 3.2.4. Targeting

**Figure 2:**
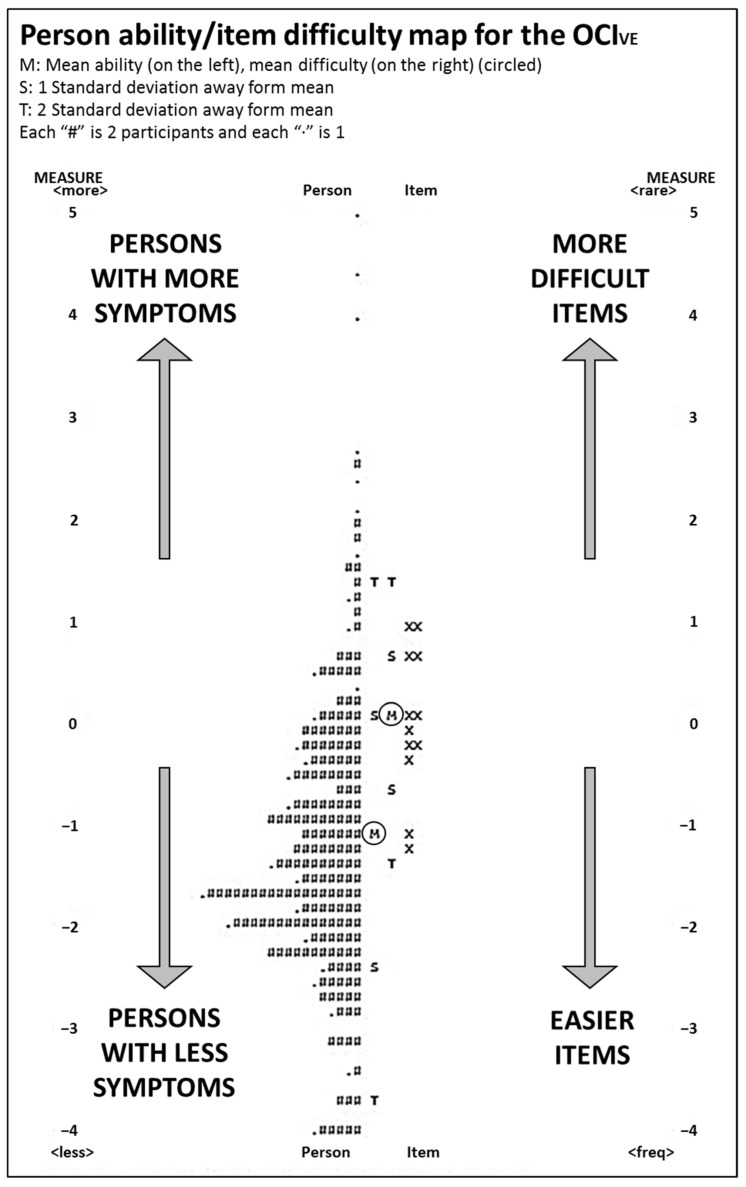
Person ability/item difficulty map for the OCI_VE_. The person (symbolized left of the vertical line) and item (symbolized to the right of the vertical line) appear in a descending order of ability and difficulty, respectively, on a scale that represents the logit value of ocular comfort, as transformed from the Rasch analysis. Responses involving higher scores of ocular discomfort (more symptomatic persons) and those items awarded lower scores (harder items) appear at the top of the scale, whereas responses involving lower scores of discomfort and easier items appear at the bottom.

Figure 2 maps person ability/item difficulty on a logit scale. The targeting value was −1.15 logits, meaning that the instrument was relatively difficult for the ability level of this sample.

#### 3.2.5. Differential Item Functioning (DIF) by Gender and Presbyopia

Differential item functioning did not vary by gender for any of the OCI_VE_ items. Just two items (item 3 “grittiness-frequency” and item 4 “grittiness-intensity”) showed DIF between the groups presbyopia and no-presbyopia (1.22 and 0.99 logits respectively), as these items could be more difficult for non-presbyopes.

To assess the psychometric properties of the OCI_VE_, we compared our Rasch analysis results against the Rasch model expectation [46], according to the quality criteria proposed by Khadka et al. [27] (Table 4).

### 3.3. English Version versus Spanish Version

For the Rasch analysis of the original English version of the OCI, after applying the exclusion criteria, the responses obtained in 216 questionnaires (mean age 40.21 ± 11.79 years; 53.24% female; 44.44% presbyopes) were used in the Andrich’s RSM analysis.

The mean OCI score obtained was 35.62 ± 12.73, on a scale of 0–100 (M = −1.07; SD = 1.32 logit scale), and the range was 0 to 62. Cronbach’s α was 0.94. Table 5 compares the main psychometric properties of the two OCI versions.

When comparing OCI and OCI_VE_, the DIF in the whole sample (N = 403 + 216 = 619) was ≤0.20 for every item. This indicates no differential functioning, as values are less than 0.50. We can, therefore, consider the versions to be equivalent.

### 3.4. Convergent Validity, Validity for Known Groups, and Repeatability

**Figure 3:**
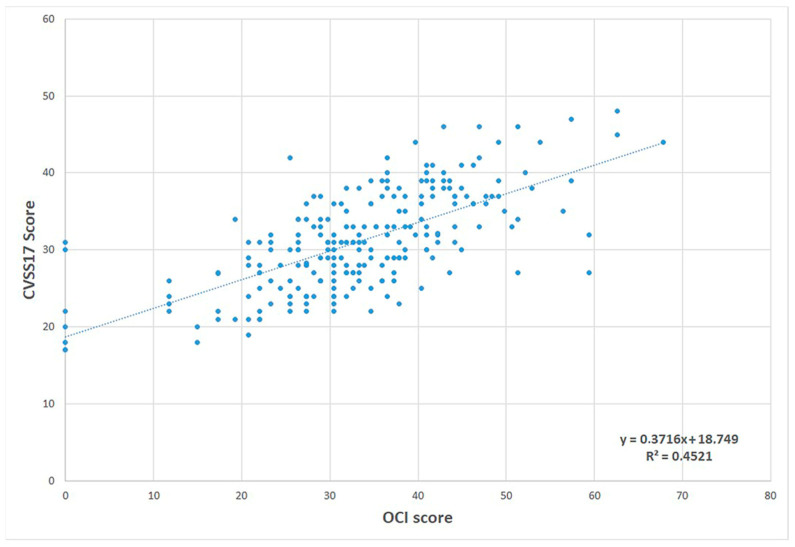
Scatterplot of correlation between the OCI_VE_ and CVSS17.

We also analyzed convergent validity for a subgroup of participants (N = 227, mean age 28.98 ± 11.71 years; 78% female; 19% presbyopes) by estimating the Spearman rho correlation between the OCI_VE_ and CVSS17; σ = 0.66 (*p* < 0.001). This correlation may be considered proof of convergent validity (Figure 3).

To analyze the convergent validity for known groups, we compared the scores obtained for each item by respondents diagnosed with dry eye and those without dry eye. Table 6 reveals that all item scores were significantly higher for the dry eye than non-dry eye group (Mann–Whitney U test; *p* < 0.001). The median of the total score (IQR) in the dry eye group was 49.09 (13.38), and the median of the total score (IQR) in the non-dry eye group was 32.04 (9.90) on a scale from 0 to 100. A significant difference was detected in the total OCI_VE_ score between the respondents with and without dry eye (Mann–Whitney U test; *p* < 0.001). Mean total scores (scale 0–100) were 51.31 ± 13.87 in the dry eye group, and 32.09 ± 7.93 in the non-dry eye group.

**Figure 4:**
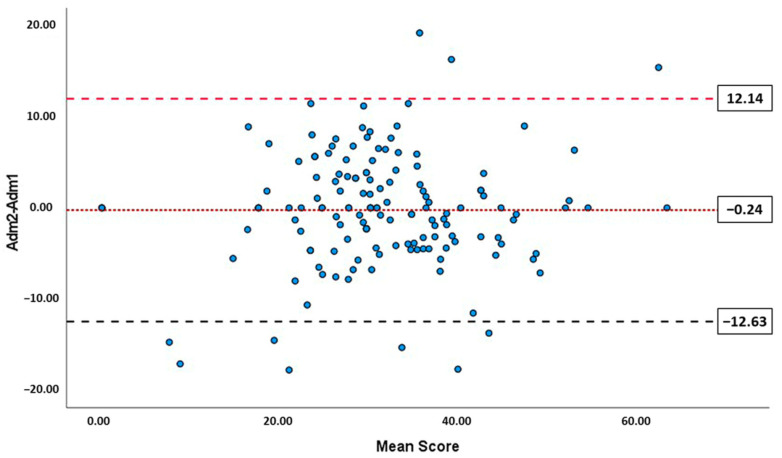
Bland–Altman plot of repeatability of OCI_VE_. The solid line indicates the mean difference (MD) between scores obtained when completing the questionnaire in two sessions. Dotted lines indicate the lower and upper 95% limits of agreement.

To analyze repeatability, 151 participants (28.05 ± 10.13 years, 76.8% female) completed the OCI_VE_ questionnaire one week after the first time. The ICC for the test–retest repeatability was 0.782 (95% confidence interval, 0.711–0.837), and the within-subject standard deviation (Sw) was 4.46. Figure 4 shows the Bland–Altman plot for OCI_VE_. The mean difference (MD) between the sessions was only −0.24. There were no significant differences between the two repeats (*p* > 0.05). The CoR (excluding four outliers showing a difference between sessions > 20 points) was ± 12.39, and the limits of agreement, including 95% of the differences (LoA) were 12.14 and –12.63 on a scale of 0–100.

## 4. Discussion

This article reports for the first time on the translation, cross-cultural adaptation, and validation of a Spanish version of the OCI questionnaire. The OCI_VE_ is available free of charge at https://concerto.cvss17.com/test/ocive (accessed on 13 November 2022) and the score is obtained automatically when it is filled in.

It is important to confirm that a translated version of an existing questionnaire consists of items that are comparable to the original items and measures the same underlying construct. We used a Rasch analysis to assess the quality of the instrument and analyzed its repeatability and validity in Spanish adults. The main advantage of a Rasch analysis is that it produces estimates on a linear interval scale rather than an ordinal scale, so it can better quantify change and offers improved statistical power [37,47]. The OCI_VE_ here showed a mean score of 34.41 ± 12.50, which is similar to that reported for the original English OCI and that obtained by Chao [29] (33.4) using the original version.

The Rasch analysis confirmed the overall good performance of the OCI_VE_ questionnaire. Its measurement accuracy was high (PSI = 3.42) indicating a good capacity to differentiate between individuals with mild or severe symptoms. This accuracy is higher than that obtained by Johnson and Murphy [26] for the original version (PSI = 2.66) and by Chao [29] for the Chinese version (PSI = 2.31).

Correlation between the OCI_VE_ and the Computer-Vision Symptom Scale CVSS17 (0.66) was greater than 0.3, which may be considered proof of a convergent validity [27]. This correlation was similar to that obtained between CVSS17 and OSDI (0.65) [48]. CVSS17 consists of two subscales: the Internal Symptom Factor (ISF) and External Symptom Factor (ESF). The latter could be a good option to assess dry eye symptoms in video display terminal (VDT) workers [48].

Although Cronbach’s α is considered by some authors to be insufficient for the analysis of the dimensionality of a scale [45,49], we decided to include it to facilitate comparisons with other scales. A Cronbach’s α value between 0.9 and 0.95 is recommended for clinical applications. This means the internal consistency of OCI_VE_ (α = 0.94) can be considered sufficient for group comparisons and clinical applications.

Our Rasch analysis of the OCI_VE_ showed a good fit to the model. Item infit and outfit statistics ranged from 0.77–1.40 and 0.75–1.16, respectively. Only item 3 (“grittiness-frequency”) showed an infit (1.40) outside the values set by Khadka (0.7–1.3), although it was still within the range considered acceptable (0.5–1.5) [27]. The difference between the ability of the respondents and the difficulty of the items was −1.15 logits. This targeting indicates the items were slightly difficult for the ability level of this sample. This frequently occurs with symptom scales, as many subjects will have only mild or no symptoms [50]. Our targeting was nevertheless better than that emerging from the Rasch analysis of the Chinese OCI version (−1.54 logits) [28].The repeatability of OCI_VE_ was good. Within-subject standard deviation was low (Sw = 4.46) and the ICC (0.782; 95%CI = 0.711–0.837) and CoR (12.39) were similar to those found for the English version (ICC 95% confidence interval = 0.81–0.91; CoR = ±13.1). It should be noted that symptoms of ocular surface disease can vary substantially, so the differences observed between repeat tests will reflect both measurement error and real person variation [51]. In our case, differences were discrete, indicated by the questionnaire’s good repeatability. This was expected as the two questionnaire sessions were only 7 days apart and because, according to the OCI’s instructions, participants answered questions about the frequency and intensity of symptoms over the past week and not only at the time they filled in the questionnaire.

In our study, the OCI_VE_’s item score and total score were able to identify patients who had been diagnosed with dry eye. In addition, the questionnaire was able to distinguish seven different levels of dry eye symptoms, which can be very useful for assessing changes in symptomatology.

According to our DIF values, female and male participants responded similarly to the questionnaire items. In contrast, responses to items 3, or “grittiness-frequency”, and 4, or “grittiness-intensity”, showed a DIF between presbyopes and non-presbyopes (1.22 and 0.99 logits, respectively), as these items could be more frequent in presbyopes.

Bradley and Massof [52] recommend comparing the psychometric properties of an adapted questionnaire with those of the original to see if the two versions perform similarly. The psychometric properties of OCI_VE_ were found here to be robust and comparable to those of the original English version [26]. In addition, there was no DIF in any item when comparing OCI and OCI_VE_. This may be taken as proof of the validity of the OCI_VE_. Accordingly, we can consider the versions equivalent, and thus, OCI_VE_ could be used in Spanish-speaking adults to grade ocular discomfort related to dry eye disease.

A limitation of our study was that the Spanish adaptation of the OCI was conducted in subjects aged over 18 years, as in the original version. However, it would be useful to have this kind of questionnaires available for younger subjects, especially because of the increase in DED detected among adolescents due to their prolonged use of digital devices.

## 5. Conclusions

OCI_VE_ shows comparable psychometric properties to the original English version, making it a valid reliable tool for measuring dry eye symptoms in Spanish adults.

## Figures and Tables

**Table 1:** Items, instructions, and response options of the original OCI and Spanish version (OCI_VE_).

	Ocular Comfort Index (OCI) Original Version (British English)	Escala Índice Confort Ocular (OCI_VE_) Translated Version to Spanish
Instructions	This questionnaire was designed to grade the comfort of your eyes. For each question, please circle your answer. Example: In the last week, how often were your eyes red? (Never) 0, 1, 2, 3, 4, ⑤, 6 (Always) There are no right or wrong answers. Do not spend too long on any one question.	Este cuestionario está diseñado para medir el confort de sus ojos. En cada pregunta, por favor, rodee su respuesta. Ejemplo: En la última semana, ¿con qué frecuencia estuvieron tus ojos rojos? (Nunca) 0, 1, 2, 3, 4, 5, ⑥ (Siempre) No hay respuestas correctas o incorrectas. No tarde mucho tiempo en responder ninguna.
1	In the last week, how often did your eyes feel dry?	En la última semana, ¿con qué frecuencia sintió sequedad en los ojos?
2	When your eyes felt dry, typically, how intense was the dryness?	Cuando sintió los ojos secos ¿cómo era de intensa la sensación de sequedad normalmente?
3	In the last week, how often did your eyes feel gritty?	En la última semana, ¿con qué frecuencia sintió sensación de arenilla en los ojos?
4	When your eyes felt gritty, typically, how intense was the grittiness?	Cuando sintió sensación de arenilla en los ojos ¿cómo era de intensa la sensación normalmente?
5	In the last week, how often did your eyes feel gritty?	En la última semana, ¿con qué frecuencia sintió sensación de arenilla en los ojos?
6	When your eyes stung, typically, how intense was the stinging?	Cuando sintió ardor o escozor en los ojos ¿Cómo era de intenso normalmente?
7	In the last week, how often did your eyes feel tired?	En la última semana, ¿con qué frecuencia notó los ojos cansados?
8	When your eyes felt tired, typically, how intense was the tiredness?	Cuando sintió los ojos cansados ¿Cómo de intenso era el cansancio normalmente?
9	In the last week, how often did your eyes feel painful?	En la última semana, ¿con qué frecuencia sintió dolor en los ojos?
10	When your eyes felt painful, typically, how intense was the pain?	Cuando sintió dolor en los ojos ¿Cómo de intenso era normalmente?
11	In the last week, how often did your eyes itch?	En la última semana, ¿con qué frecuencia le picaron los ojos?
12	When your eyes itched, typically, how intense was the itching?	Cuando le picaron los ojos ¿Cómo de intenso era el picor normalmente?
Response	Never (0), 1, 2, 3, 4, 5 (6) Always Never had it (0), 1, 2 3, 4, 5 (6) Severe	Nunca (0), 1, 2, 3, 4, 5 (6) Siempre Nunca lo he tenido (0), 1, 2 3, 4, 5 (6) Muy intenso

**Table 2:** Rasch Fit Statistics and Item Measure for OCI_VE_.

Item Number	Item	Infit (MNSQ)	Outfit (MNSQ)	Measure (Logits)
1	En la última semana, ¿con qué frecuencia sintió sequedad en los ojos?	1.00	0.99	−0.27
2	Cuando sintió los ojos secos ¿cómo era de intensa la sensación de sequedad normalmente?	0.77	0.75	−0.18
3	En la última semana, ¿con qué frecuencia sintió sensación de arenilla en los ojos?	1.40	1.16	1.00
4	Cuando sintió sensación de arenilla en los ojos ¿cómo era de intensa la sensación normalmente?	1.28	1.12	0.97
5	En la última semana, ¿con qué frecuencia sintió ardor o escozor en los ojos?	0.87	0.82	0.01
6	Cuando sintió ardor o escozor en los ojos ¿Cómo era de intenso normalmente?	0.80	0.77	0.03
7	En la última semana, ¿con qué frecuencia notó los ojos cansados?	0.91	0.96	−1.29
8	Cuando sintió los ojos cansados ¿Cómo de intenso era el cansancio normalmente?	0.82	0.88	−1.04
9	En la última semana, ¿con qué frecuencia sintió dolor en los ojos?	1.19	1.03	0.66
10	Cuando sintió dolor en los ojos ¿Cómo de intenso era normalmente?	1.16	1.08	0.63
11	En la última semana, ¿con qué frecuencia le picaron los ojos?	1.08	1.04	−0.37
12	Cuando le picaron los ojos ¿Cómo de intenso era el picor normalmente?	0.91	0.91	−0.15

**Table 3:** OCI_VE_ levels of performance (symptoms severity).

	OCI_VE_ Levels of Performance						
	Level 1	Level 2	Level 3	Level 4	Level 5	Level 6	Level 7
Score range	≥0 to ≤29	>29 to ≤36	>36 to ≤43	>43 to ≤51	>51 to ≤59	>59 to ≤70	>70 to ≤100

**Table 4:** Psychometric Properties of OCI_VE_.

Parameter	Rasch Model Expectation	OCI_VE_
Number of items	-	12
Person separation index PSI (reliability)	>2.0 (>0.80)	3.42 (0.90)
PCA. Raw variance explained by measure	>50	67.3
Number of items with infit outside range 0.7 to 1.3	0	1
Number of items with outfit outside range 0.7 to 1.3	0	0
Number of items with DIF-gender > 0.5 logits	0	0
Number of items with DIF-presbyopia > 0.5 logits	0	2
Targeting	≥−1.0	−1.15

**Table 5:** Psychometric Properties of the Spanish and English OCI Versions.

Parameter	OCI_VE_	OCI
Number of items	12	12
Person separation index PSI (reliability)	3.42 (0.90)	3.22 (0.91)
PCA. Raw variance explained by measure	67.3%	65%
Number of items with infit outside range 0.7 to 1.3	1	0
Number of items with outfit outside range 0.7 to 1.3	0	1
Number of items with DIF-gender > 0.5 logits	0	0
Number of items with DIF-presbyopia > 0.5 logits	2	0
Targeting	12	12

**Table 6:** Median (IQR) Scores for 12 Questions Awarded by Respondents with and without Dry Eye.

Item Number	Item	Dry Eye (n = 53)	No Dry Eye (n = 53)	*p*-Value
1.	Dryness-Frequency	5 (2)	1 (3)	
2.	Dryness-Intensity	5 (1)	2 (2)	
3.	Grittiness-Frequency	4 (3)	0 (2)	
4.	Grittiness-Intensity	4 (2)	0 (2)	
5.	Eye stinging-Frequency	4 (3)	1 (2)	<0.001
6.	Eye stinging-Intensity	4 (2)	1 (3)	
7.	Eye tiredness-Frequency	5 (2)	2 (1)	
8.	Eye tiredness-Intensity	5 (2)	2 (2)	
9.	Eye pain-Frequency	2 (3)	0 (2)	
10.	Eye pain-Intensity	4 (4)	0 (2)	
11.	Eye itchiness-Frequency	4 (3)	2 (2)	
12.	Eye itchiness-Intensity	3 (3)	2 (1)	

## Data Availability

Not applicable.
